# Scurvy, a not obsolete disorder: Clinical report in eight young children and literature review

**DOI:** 10.1515/med-2024-1086

**Published:** 2025-09-25

**Authors:** Alessandra Di Nora, Maria Carla Finocchiaro, Francesco Pizzo, Trobia Gian Luca, Maria Elena Cucuzza, Antonella Di Stefano, Serena Spampinato, Silvia Marino, Martino Ruggieri, Piero Pavone

**Affiliations:** Section of Pediatrics and Child Neuropsychiatry, School of Specialization in Pediatrics, Department of Clinical and Experimental Medicine, University of Catania, 95124, Catania, Italy; Pediatrics and Pediatric Emergency Room, Cannizzaro Emergency Hospital, 95126, Catania, Italy; Department of Clinical and Experimental Medicine, Unit of Infectious Diseases, University of Catania, ARNAS Garibaldi Hospital, I-95122, Catania, Italy; Unit of Catania, Institute for Biomedical Research and Innovation, National Council of Research, Catania, Italy

**Keywords:** l-ascorbic acid, scurvy, vitamin C deficiency, gingival bleeding, swollen gum

## Abstract

**Background:**

Vitamin C is a key to many important functions. It stimulates the immune system by protecting humans from infections and shows notable anti-viral and anti-inflammatory properties. With the antioxidative properties it acts against free radicals and cellular aging and prevents tumors. It is also involved in the synthesis of collagen, a structural protein that is essential for the formation of connective tissue as epidermis, muscle, bone, cartilage, etc. Vitamin C promotes the absorption of iron contributing to the production of red blood cells and the synthesis of hemoglobin. Scurvy is a nutritional disorder caused by low vitamin C levels which manifests with varied symptoms affecting multiple organ systems. Vitamin C also known as l-ascorbic acid, is a water-soluble nutrient and is a necessary element as the humans are unable to synthesize it. Vitamin C has an important role in the biochemical reactions of connective tissue synthesis. Presenting manifestations include malaise, gingival bleeding, impaired wound healing, perifollicular hemorrhage, dry hair and brittle nails, iron deficiency, muscle and joint pain, pulmonary hypertension, and other symptoms. The persistent reduced supply of vitamin C in the absence of treatment is cause of a severe progressive worsening of the clinical conditions. The disorder is uncommonly reported in high social level countries and in mentally wellbeing children.

**Methods:**

Herewith, we report case-series of eight children with scurvy diagnosed in two Pediatric Hospitals in Catania, Italy “Policlinico G. Rodolico” and “Cannizzaro” in the last 2 years, October 2021–October 2023. In addition, a systematic literature review of 126 articles with 253 cases of scurvy including age, sex, main clinical manifestations, and eventual presence of neurodevelopmental disorders is reported. Main characteristic of vitamin C and negative effects of its lack with clinical manifestations, diagnosis, treatment, and prognosis are also referred.

**Results:**

Malnutrition, gastrointestinal, and neurological disorders, are the associated predisposing factors. In the present case-series, to the higher incidence of scurvy compared to others Italian Regions may have contributed an erroneous old prejudice of parents who refuse to give citrus fruits to young children as lemon and oranges may cause cystitis as well severe toxicity when mixed with milk.

**Conclusion:**

This study aims to alert on the scurvy as a possible cause of childhood disorder also in well industrialized regions, and to offer diagnostic tools for identifying subjects suffering from this illness.

## 1 Introduction

Scurvy is a clinical syndrome caused by vitamin C deficiency. It is a disorder globally diffused and mainly observed in countries with poor social conditions, in malnourished people, in individuals affected by various gastrointestinal and neurobehavioral disturbances which may prevent the absorption of important nutrients such as vitamin C [1–6]. Scurvy has been known since ancient times. During the period of Renaissance running from fourteenth to sixteenth centuries, several epidemic episodes of scurvy among sea voyagers were reported and it was noted that supply of oranges and lemons had a notably favorable clinical outcome on the affected individuals [7,8]. Classic clinical and pathological features of infantile scurvy were reported by Thomas Barlow in 1883 and an important insight in the prevention of scurvy was due to Alfred Hess who noted that pasteurization reduced the antiscorbutic effect of milk thus favoring the increase of cases of scurvy and suggested supplementation of fresh fruit and vegetable juices to prevent scurvy [8].

The prevalence of the disorder differs according to the age, lifestyle, nutritional habit, and associated underlying disorders. It has been reported ranging from 7.1% as registered in United States to 73.9% in some area of northern India [1]. In a study conducted by Ravindran et al. [3] in 2,668 individuals aged 60 years and over, sex and season standardized, prevalence of vitamin C deficiency was reported in 73.9% (95% confidence interval [Cl] 70.4, 77.5) subjects in North India and in 47.7% (95% Cl 42.5, 48.9) in 2,970 from South India. According to the authors [3] the deficit of vitamin C was more prevalent in men, in people with increasing age, users of tobacco, and biomass fuels, as well in people with poor nutrition, and with lower intakes of dietary vitamin C. They report data of scurvy registered in the UK and in North America where the disorder in low income population was found to affect around 1 on 5 men and 1 on 9 women [3].

Vitamin C, also known as l-ascorbic acid is not synthesized by humans who lack the active form of the enzyme l-gulonolactone oxidase required for synthesizing ascorbic acid and therefore it must be obtained through the diet in the form of fruits and vegetables. Vitamin C is a water- soluble vitamin which shows antioxidant function and is an essential co-factor for collagen biosynthesis, carnitine, catecholamine metabolism, and dietary iron absorption [1–3]. Common clinical manifestations of scurvy include gingival bleeding, skin discoloration, petechial rash, and ecchymosis caused by defective collagen synthesis of blood vessel walls, perifollicular hemorrhage, impaired wound healing, bone and muscle pain, and other symptoms [1–5,9]. Scurvy is a well-known disorder but still present in childhood clinical practice.

Herewith, we summarize the clinical results observed on eight children affected by scurvy admitted in two hospitals in Catania, “Policlinico G Rodolico” and “Cannizzaro” in the course of the last 2 years (October 2021–October 2023). In addition, results of a systematic review of 126 articles with 253 cases of scurvy including age, sex, main clinical manifestations, and eventual neurodevelopmental disorders were summarized. Vitamin C characteristics and the effects of lack presenting with clinical features, diagnosis, treatment, and prognosis are discussed as to better understand this disorder. Scurvy is an almost historic disorder but still not vanished in good well-being population.

### 1.1 Case series

**Figure 1:**
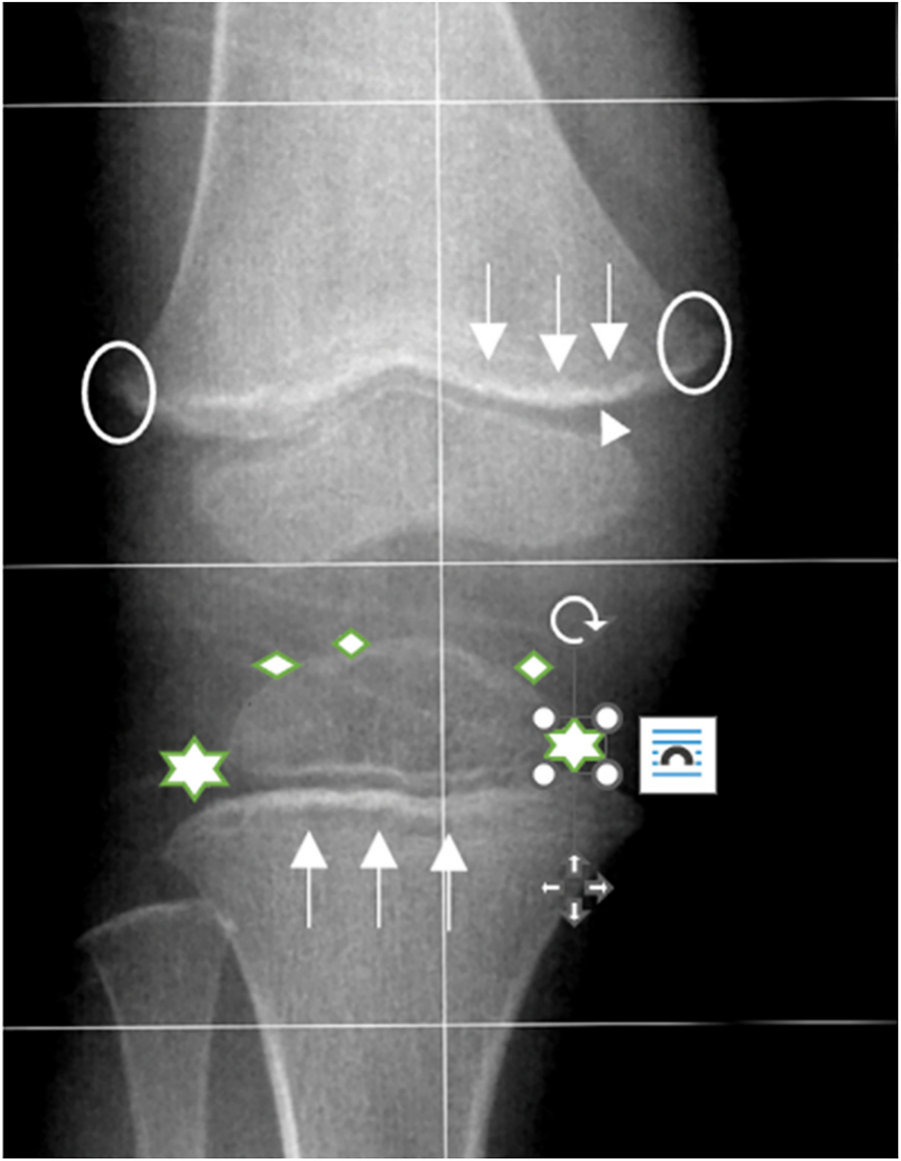
Scurvy in 3-year-old boy with skeletal pain (ID-08). Antero-posterior right knee radiography shows the classic radiographic signs described in literature: Dense zone of provisional calcification at edge of metaphysis (white line of Frankel) (head arrows), transverse radiolucent metaphyseal line (Trümmerfeld zone) (white arrows), circular calcification surrounding the osteoporotic epiphyseal center of ossification (Wimberger ring sign), and beaklike metaphyseal excrescences (Pelkan spurs, corner or angle sign) (white circles) [5].

Eight children were admitted to the Pediatric Department with different symptoms, united by lower limbs pain and refusal to walk who were treated at home with non-steroidal anti-inflammatory drugs, without benefit. In Table 1, we summarize the age at diagnosis, gender, clinical manifestations at admission, diagnostic workup, food intake, and neurodevelopmental profile of each of the eight children. At diagnosis the age ranged from 17 months to 12 years. The gender was prevalent in male M6/F2. The symptoms singularly or in association presented by the children were the following: limping n.5, hyperkeratosis pilar n.4, gingival bleeding n.3, gingival swelling n.2, skeletal pain n.2, anemia n.2, frequent fever, and knee hemarthrosis n.1. In two patients with skeletal and muscles pain, the radiological examinations revealed the typical signs of scurvy (Figure 1). In the other patients who had the radiological examinations no anomalies were reported. Two patients presented neurobehavioral disorders, the other children were neurologically normal. Personal history showed an absolute lack of nutrients with vitamin C content and absence of citrus fruits supply (ID-01, ID-02, ID-03, ID-05, and ID-08). The diagnosis was confirmed by low content of vitamin C with value in all the cases <0.3 mg/dL. Two children showed anemia with Hb values between 10 and 11 g/dL. Scurvy treatment consisted of 100–300 mg daily according to the age of children. After treatment a rapid improvement of the symptoms were obtained within 2 weeks. After treatment, the parents were advised to introduce citrus fruits in the diet daily. To our knowledge no further complications were reported by the parents.

**Table 1:** Present case series

Summary of the main clinical results in children affected by scurvy						
ID patient	Age at diagnosis	Sex	Clinical manifestations	Diagnostic tools	Selective food intake	Neurodevelopmental disorders
ID-01	2 years	M	Fever, limping, hyperkeratosis pilar	Serological, clinical signs, X-ray, and magnetic resonance	Routine home-based diet devoid of fruit and vegetables	No
ID-02	3 years	M	Limping, gingival bleeding, and swelling	Serological and clinical signs	Routine home-based diet devoid of fruit and vegetables	No
ID-03	17 months	M	Skeletal pain, gingival bleeding	Serological and clinical signs	Milk and cereals	No
ID-04	2 years	M	Limping, hyperkeratosis pilar	Serological, X-ray	Routine home-based diet devoid of fruit and vegetables	No
ID-05	3 years	M	Limping, hyperkeratosis pilar, anemia	Serological, X-ray and magnetic resonance	Routine home-based diet devoid of fruit and vegetables	No
ID-06	12 years	F	Knee hemarthrosis, gingival swelling	Serological	Milk and biscuits, prevalently	Autism spectrum disorder (ASD)
ID-07	7 years	M	Limping, hyperkeratosis pilar, anemia	Serological	Avoiding restrictive food intake disorder (ARFID)	ARFID
ID-08	3 years	F	Skeletal pain, gingival bleeding	Serological, X-ray	Routine home-based diet devoid of fruit and vegetables	No

Note: ARFID: avoidant/restrictive food intake disorder.

## 2 Systematic review

### 2.1 Materials and methods

**Figure 2:**
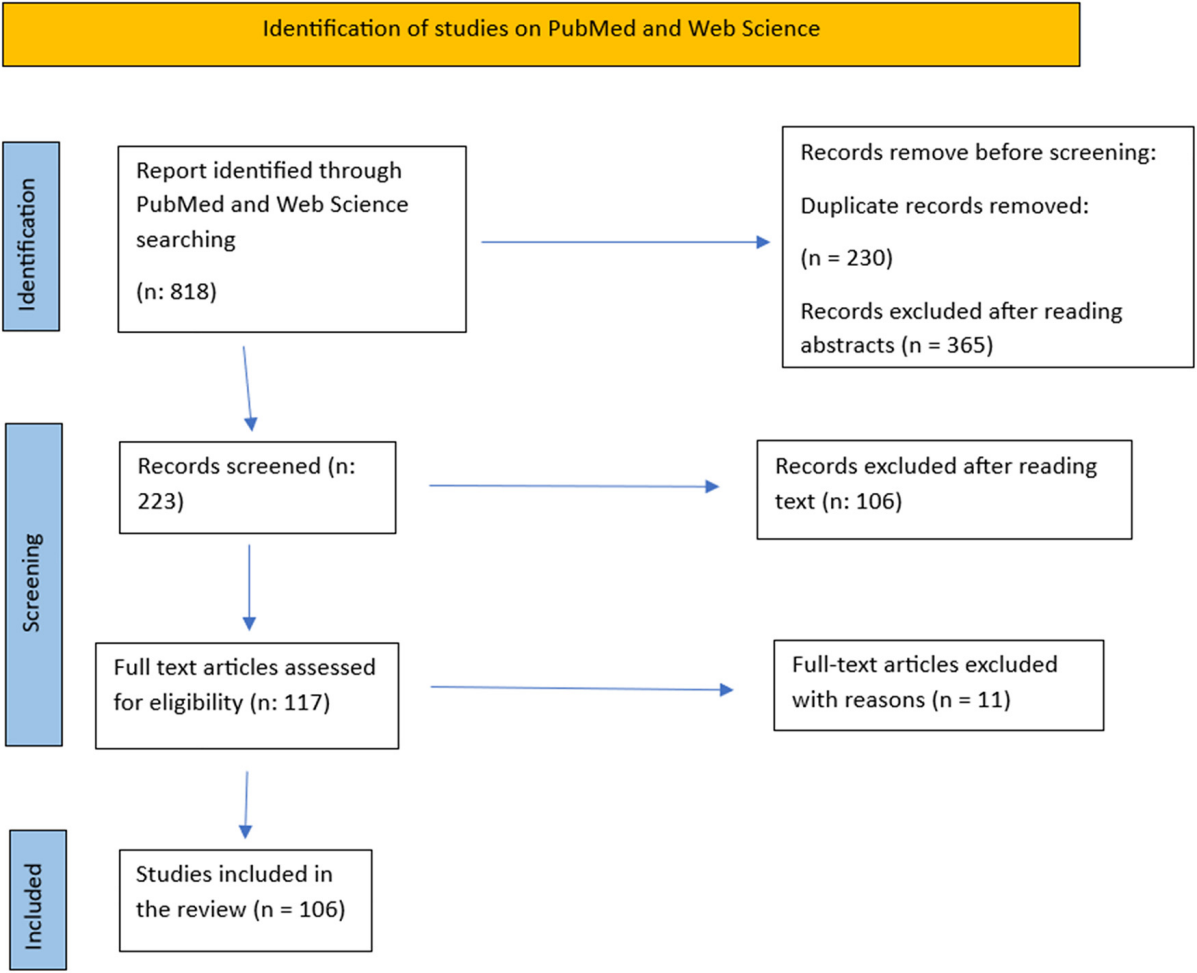
PRISMA method.


This systematic review was conducted according to the guidelines of the Preferred Reporting Items for Systematic Reviews and Meta-Analyses (PRISMA) [10]. Two medical electronic databases (PubMed and Web of Science) were searched by two authors (A.D. and F.P.). The research string used was “Scurvy,” “Vitamin C deficiency,” “Moeller’s disease,” “Cheadle’s disease,” “Scorbutus,” “Barlow’s disease,” “Hypoascorbemia,” “Lack of vitamin C” AND “pathology” OR “etiology” OR “etiopathogenesis” OR “pathophysiology” OR “risk factors” OR “associated factors” OR “predisposing factors”. In total, *n* = 818 articles were found. Two reviewers, once the initial results were collected, analyzed the titles and abstracts screening the following inclusion criteria: studies of any level of evidence reporting clinical results published in the last 22 years (2000–2022). Comparative, cross-sectional, retrospective, prospective, and survey studies, case series and case reports on pediatric population were particularly selected. All the articles written in languages other than English were excluded. All articles that dealt with different topics, had poor scientific methodology, or were without an accessible abstract were excluded. Also, duplicates were excluded and all those articles that did not match of the inclusion criteria were ruled out. The full texts of the remaining articles were read in depth by two reviewers to better assess the content of the studies: demographic data, clinical manifestations, and neurodevelopment profile. The extracted data have been synthesized (Figure 2).

**Informed consent:** Written informed consent has been obtained from the patient to publish this article.
**Ethical approval:** The study was conducted according to the guidelines of the Declaration of Helsinki.

## 3 Results

The initial search yielded 818 results; 230 articles were excluded as they were duplicates. Another 365 articles were excluded following the reading of titles and abstracts. Reading the full text of the 223 remaining articles, 106 were excluded after reading the text. At the end of the selection, 106 articles were included in this systematic review (Figure 2).

A total number of 253 patients were described within the 106 selected studies. Details on the 106 selected items of 253 subjects, including number of patients for each author, age, sex, main clinical manifestations, and neurodevelopmental conditions are reported in Table 2 [11–116]. The age of children ranged with a minimum age of 5 months and a maximum age of 16 years. The most common age group was the age between 24 and 36 months. The gender prevalence was male/female (M/F) 113:28 in patients of this study and gender prevalence M/F 5:1 in children of our cohort. There was a wide prevalence of children with neurological disorders with ASD as the main representative disease followed by children with neurodevelopmental disorders aside the ASD. Children with scurvy showed a varied spectrum of clinical features: refusal of walk, irritability, malaise, gingival bleeding, gum swelling, petechial hemorrhages, tenderness, and swelling at lower extremities were the most common features. Cutaneous manifestations included petechiae, ecchymoses, and hyperkeratosis. Palpable purpura and widespread ecchymoses were frequently observed mimicking vasculitis. Gums may present swollen, loosen, and bleed on slight pressure in a majority of patients. Musculoskeletal manifestations such as limping, arthralgia, and myalgia often represent the first clinical manifestation requiring medical attention, with or without evident cutaneous manifestations.

**Table 2:** Scurvy: Main clinical data from 106 literature reports and 253 pediatric subjects

	Patient (*n*)	Age	Sex	Clinical manifestations	Selective food intake	Neurodevelopmental disorders
Narchi and Thomas [11]	1	1.5 years	M	Gum swelling and tenderness, skeletal involvement	Milk and biscuits	No
Weinstein et al. [12]	1	9 years	F	Musculoskeletal disorders, oral manifestations	Chocolate and milk	Developmental delay
Riepe et al. [13]	1	1.3 years	M	Musculoskeletal disorders, cutaneous, and oral manifestations	Cow’s milk and oatmeal	Not reported
Gorman et al. [14]	1	0.5 years	M	Cutaneous and oral manifestations	Not reported	No
Ahuja and Karande [15]	1	1.5 years	M	Edema over the scalp, petechiae over the chin, swelling around eyes	Milk	No
Akikusa et al. [16]	1	9 years	M	Gingival hypertrophy, weak of hip flexion and extension, hepatic enlargement	Bread, milk, and chocolate	No
Bingham et al. [17]	1	16 years	M	Gum swelling and leg cramps, ecchymotic lesions on his lower extremities, gingival hypertrophy	Very restrictive diet	No
Ratageri et al. [18]	3	1.4 years, 0.9 years, 1 years	M, M, M	Case 1: Thigh tenderness, gingival swelling; Case 2: Thigh tenderness, hyperpigmentation, gingival swelling; Case 3: Hyperpigmentation	All cases exclusive breast-feeding	Developmental delay in all
Rosati et al. [19]	1	8 years	F	Diffused petechial hemorrhages, ecchymosis of the buttocks, and swollen, friable gums	Not reported	No
Verma et al. [20]	1	3 years	M	Proptosis of left eye, swollen gums, tenderness and swelling of the thighs, Pelkan spur	Milk and rice	No
Burk and Molodow [21]	1	2 years	M	Lower extremities pain with bruising, malaise, gingival swelling, skin xerosis, hair corkscrewing, ecchymoses, metaphyseal bands	Home-routine devoid of fruit and vegetables	No
Willmott and Bryan [22]	1	9 years	F	Gingival swelling and bleeding, leg’s petechial rash, swollen extremities and knees	Ketogenic diet (since 4–5 years)	Delay
Kumar et al. [23]	1	5 years	F	Gingival bleeding, swelling of left ankle, petechiae and hyperkeratosis on lower limbs, white lines of Fraenkel	Adequate diet in proteins and calories	No
Mawson [24]	1	9 years	M	Skin rash; gingival swelling, bleeding, and bone disease related to subperiosteal bleeding; dense zone of calcification at the margins of the growth plate with lucent line	Restricted diet	Autism
Vitale et al. [25]	2	8 years, 2 years	M, F	Case 1: Swollen knees, petechiae, follicular hyperkeratosis, diffuse ecchymosis, gingival swelling, and bleeding; Case 2: Increasing bilateral knee swelling, and marked oedematous gums with bleeding	Biscuits and milk	1: Delay;2: No
Popovich et al. [26]	1	1.66 years	M	Musculoskeletal tenderness, oral manifestations, microcytic anemia	Meat and rice	No
Bursali et al. [27]	1	1.33 years	M	Swelling and tenderness of limbs, gingival swollen, and bleeding	Home-routine devoid of fruits and vegetables	No
Bacci et al. [28]	1	2 years	F	Gingival swelling and bleeding, swelling of knee, muscular frailly	Milk, biscuit	No
Ghedira Besbes et al. [29]	2	2.3 years, 5 years	M, M	Case 1: Musculoskeletal pain, bilateral knee pain, bleeding of the gums; Case 2: Bleeding of the gums	All cases milk exclusively	Developmental delay in both
Solanki et al. [30]	1	10 years	M	Gingival swelling and bleeding, perifollicular hyperkeratosis	Restrictive diet	Delay
Cole et al. [31]	1	10 years	M	Bruising, swelling and ecchymosis of lower extremities, gingival bleeding and swelling	Meat and chocolate	Autism
Estienne et al. [32]	1	2.5 years	M	Gingival bleeding and swelling, perifollicular hemorrhages, plantar desquamation and distal edema	Milk	No
Valentini et al. [33]	1	1.1 years	M	Bruises and swelling of lower limbs, purpuric lesions, Frankel’s line, Wimberger’s sign	Breast-feeding and artificial milk only	No
Brennan et al. [34]	1	5 years	M	Muskuloskeletal pain with knee tenderness, fatigue, abdominal pain	Restrictive diet, devoid of fruits and vegetable	
Dey et al. [35]	1	9 years	M	Several bruises and petechiae, gingival swelling and bleeding	Milk and chocolate	Autism
Codreanu et al. [36]	1	4 years	M	Chronic glossitis, anemia	Eggs, biscuits, chocolate and milk	No
De Cock et al. [37]	1	3 years	M	Lower limb weakness	Selective diet	Autism
Gupta et al. [38]	1	6 years	F	Swelling of bilateral shoulders and bilateral knees, oral ulcers	Not reported	Cerebral palsy
Niwa et al. [39]	1	6 years	M	Dermal spotted hemorrhagic foci, petechiae, gingival bleeding and swelling, oral mucosal bleeding	Restrictive diet	Autism
Saha et al. [40]	1	5 years	M	Bilateral proptosis of both eyes, gingival bleeding	Rice and milk	Cerebral palsy
Noordin et al. [41]	1	4.5 years	M	Gingival swelling, knee and ankle joint disorders and osteoporosis	Restricted diet	No
Duvall et al. [42]	1	9 years	M	Limping, bleeding gums, dry lips, and sunken eyes	Not reported	Autism
Gongidi et al. [43]	1	5 years	M	Muskuloskeletal pain, gingival swelling	Chocolate milk	Autism
Rumsey and Rosenberg [44]	1	8 years	M	Gingival swelling and bleeding, joint warmth and swelling, intraarticular effusion	Restrictive diet	Autism
Kitcharoensakkul et al. [45]	3	5 years, 5 years, 5 years	F, M, M	Case 1: Joint swelling, gingival swelling, and bleeding; Cases 2–3: Bleeding of the gums, joint swelling, and rashes	Home diet devoid of fruits and vegetables	1 and 2: No delay; 3: Autism
Barbera et al. [46]	1	16 years	M	Musculoskeletal symptoms, petechiae, and ecchymoses of extremities	Cheese and pizza	Autism
Harknett et al. [47]	1	9 years	M	Leg pain and swelling, gingival bleeding	Oatmeal and soy milk	Autism
Sobotka et al. [48]	1	10 years	M	Non-pruritic, non-painful, petechial rash on his lower legs, easy bruising and bleeding	Very restricted diet	Autism
Khan et al. [49]	1	8 years	M	Gum swelling and bleeding, low-grade fever, and a maculopapular rash in bilateral upper and lower extremities	Restrictive diet	Autism
Gulko et al. [50]	4	8 years, 7 years, 6 years, 5 years	M, M, M, M	All cases bleeding gums, petechiae on limbs and trunk, limping	Restrictive diet and feeding aversion	Autism
Polat et al. [51]	1	6 years	F	Purpura diffused on the whole body, gingival bleeding, and nose bleeding; generalized osteopenia	Not reported	No
Alqanatish et al. [52]	1	12 years	M	Lower extremities follicular purpura, gingival swelling	Exclusively milk-fed	Developmental delay
Vitoria et al. [53]	1	0.92 years	M	Generalized and marked osteopenia, fractures	Exclusive use of almond beverages in the first year	No
Hafez et al. [54]	1	2.5 years	M	Bleeding gingival mucosa, follicular hyperkeratosis with perifollicular hemorrhages	Chocolate and crackers	Developmental delay
Ma et al. [55]	3	4 years, 11 years, 11 years	M, M, M	Case 1: Hemorrhages at the knees and gums; Case 2: Gingival swelling and bruising, bleeding along the distal thigh and proximal tibia; Case 3: Swollen gums and ecchymosis over the left knee	Selective diet	Autism (1, 2, 3)
Tchaou et al. [56]	1	2 years	M	Swelling and bleeding of the gums, and of the thighs and knees	Meat and milk mainly	No
Seya et al. [57]	1	5 years	M	Painful knees and difficulty walking	Selective diet	No
Bouaziz et al. [58]	1	4 years	F	Shoulder bruising and swelling, general osteopenia	Diet: Only milk	No
Aziz et al. [59]	1	0.92 years	M	Eyelid edema, oral manifestations	Not reported	No
Uda et al. [60]	1	3 years	M	Swelling knees, petechiae, gingival bleeding	Not reported	No
Jacobsen and DeNiro [61]	1	16 years	M	Edema, ecchymosis, and arthralgias of lower extremity; fatigue, abdominal discomfort; gingival swelling and bleeding; large ecchymoses on the thighs, ankles, and feet and scattered petechiae	Mainly crackers and peanut butter	Autism
Kaur and Goraya [62]	1	1.75 years	F	Swelling of the right leg, rachitic rosary, gingival bleeding, white dense line of metaphyseal calcification (Fraenkel’s line)	Not reported	Developmental delay
Brambilla et al. [63]	1	3 years	F	Erythematous and itchy rash, left thigh swelling, diffuse petechiae	Very selective diet	No
Kakade et al. [64]	1	10 years	M	Bluish-red, soft, tender, hemorrhagic gingival, limping, pain extremities and microcytic anemia	Restrictive diet	No
Saavedra et al. [65]	1	4 years	M	Diffuse petechiae on the lower limbs, as well as spontaneous bruising on the knees	Restricted diet	Autism
Kinlin et al. [66]	1	10 years	M	Ankle swelling, parafollicular hyperkeratosis, ecchymosis over the medial aspect of the right ankle, and subtle gingival changes	Restricted diet	Autism
Harikrishnan and Suma [67]	2	3 years, 6 years	M, M	Case 1: Fullness and tenderness of knee joints, diffuse osteopenia, irregular thickened white line at the metaphysis of femurs, and ring epiphysis; Case 2: Bleeding of the gums, osteopenia with lucent metaphyseal bands	Case 1: Milk; Case 2: Biscuits and noodles	1: Developmental delay; 2: Autism
Küçükaydın et al. [68]	1	7 years	M	Swollen and bleeding gum, follicular hyperkeratosis with perifollicular purpura at the lower extremities, and soft tissue swelling of both knees	Yogurt soup, chocolate, and wheat bread	Autism
Ceglie et al. [69]	3	2.5 years, 5 years, 2 years	M, M, M	Case 1: Diffused petechiae, gum swelling, and bleeding; Case 2: Gingival bleeding and hypertrophy; Case 3: Limping and swelling of the right knee	Case 1: Refusal fruits and vegetables; Case 2: Selective diet; Case 3: Selective diet	Normal development in all
Rafee et al. [70]	1	14 years	M	Sinus tachycardia, bleeding of nose and gums	Selective diet	Autism
Ichiyanagi et al. [71]	1	3 years	M	Pulmonary hypertension, gingival bleeding, humeral fractures	Not reported	Autism
Lund et al. [72]	1	3 years	F	General osteopenia and musculoskeletal tenderness	Not reported	No
Nastro et al. [73]	1	4 years	M	Petechial rash, bleeding gingival, mild gingivitis	Picky eater	No
Ozcan et al. [74]	1	10 years	F	Fanconi anemia, gum enlargement, petechiae, purpura, and hyperpigmented lesions on the trunk and extremities	Not reported	No
Hahn et al. [75]	1	5 years	F	Bleeding of the gums, small pink flat papules on lower left leg, bilateral lower extremity pain, limping, knee and ankle pain	Selective diet	No
Dean et al. [76]	1	6 years	M	Gingival bleeding, bone edema	Severe restrictive diet	No
Kothari et al. [77]	2	7 years, 8 years	M, M	Case 1: Erythematous, hemorrhagic, swollen gingiva, contractures in the knees, rash on the left arm, consistent with parafollicular keratosis; Case 2: Severe gingivitis, and dental abscess; erythematous and edematous gingiva, generalized calculus build up, and decay involving the maxillary right primary second molar	Picky eater	Autism (1, 2)
Hahn et al. [78]	3	8 years, 6 years, 4 years	M, M, M	Case 1: Leg pain; Case 2: Iron deficiency anemia, leg pain; Case 3: Iron deficiency anemia	Case 1: Cheerios, Frosted Flakes, Goldfish, pretzels, and milk; Case 2: Milk, chicken nuggets, and Kit Kat candy bars; Case 3: Milk, yogurt, apple, banana, cookies, candy, and French fries	Autism
Benezech et al. [79]	1	14 years	M	Pancytopenia, inflammatory gingival swallowing, follicular inflammation of the legs mimicking purpura, and swelling of the right knee joint	Avoidant/restrictive food intake disorder	No
Boone et al. [80]	1	2 years	F	Gingival bleeding, abdominal pain, leg pain, fever	Almost exclusively starches	No
Burhop et al. [81]	1	11 years	M	Gingival hyperplasia and hematomas	Restricted diet	Autism
Gallizzi et al. [82]	1	3 years	M	Gums hypertrophy and bleeding purpura, musculoskeletal pain	Restrictive diet	No
Gupta et al. [83]	1	4 years	M	Bilateral lower limb pain, bleeding of the gums	Home routine devoid of vegetables and fruits	No
Chalouhi et al. [84]	3	3 years, 3.5 years, 3 years	M, F, F	Case 1: Conjunctival hyperemia and bleeding from the eye and erythematous gums; Case 2: Gingival hypertrophy, intermittent limping; Case 3: Lower limb pain, dry skin, inflammatory hair follicles	Selective diet (1, 2, 3)	No
Cheah et al. [85]	1	7 years	F	Right eye proptosis and chemosis, gums bleeding	Restrictive diet	Developmental delay
Fortenberry et al. [86]	5	7 years, 10 years, 10 years, 6 years, 14 years	M, M, M, M, M	Case 1: Gingival bleeding and petechial rash; Case 2: Bilateral ankle swelling and mild bruising along the calves; Case 3: Painful mouth lesions on the soft palate, frequent nosebleeds, petechial rash and redness around the hair follicles on his legs, mandibular gum swelling; Case 4: Microcytic anemia; Case 5: Petechial rash, lower extremity bruising	Restrictive diet	Autism in all the cases
Schwetje et al. [87]	2	6 years, 6 years	M, M	Case 1: Gingival bleeding, swelling of both legs; Case 2: Recurrent infections, oral bleeding, vomiting, anemia, gastric bleeding	Not reported	1: Cerebral palsy; 2: No
Luckow and Thomas [88]	1	5 years	M	Erythematous pinpoint macular rash, bleeding gums	Selective diet	Autism
Diab Shehade et al. [89]	1	3 years	F	Leg limping, perifollicular petechiae, bleeding and swelling gums, anemia	Restrictive diet	No
Musa et al. [90]	1	4 years	M	Bilateral lower limb pain and refusal to walk	Restrictive diet	Developmental delay
Rubino et al. [91]	3	4 years, 3 years, 1.6 years	M, M, M	Skeletal and oral manifestations	Restrictive diet	1: Autism, 2: No, 3: No
Pan et al. [92]	9	The average age was 7 years (range 3–13 years)	7 M, 2 F	One presented with bone pain, four presented with limb swelling, seven had unilateral and two had bilateral leg symptoms, five presented with inability to walk, six demonstrated skin changes with ecchymosis or petechiae, three presented with gingival bleeding	Abnormal diet	Five had autism, two had neurological disorder; two had been born premature; two had psychiatric disorder
Likhitweerawong et al. [93]	1	3 years	M	Arthralgia and anaemia	Restrictive diet	Autism
Manzie et al. [94]	1	12 years	F	Bleeding/bruising, anaemia and gingival hyperplasia	Restrictive diet	Autism
Jain et al. [95]	1	4 years	F	Fever and bilateral knee joint pain	Restrictive diet	Developmental delay
Murakami et al. [96]	2	3 years, 3 years	M, M	Gingival bleeding, skeletal manifestations	Restrictive diet	1: No, 2: Developmental delay
Abe et al. [97]	1	7 years	M	Reversible pulmonary hypertension and right-sided heart failure polyarthralgia, gingival hyperplasia with ecchymosis, and fatigue	Restrictive diet	Developmental delay
Ahmad et al. [98]	1	5 years	F	Skeletal manifestations	Ketogenic diet	Developmental delay
Tran and Yuan [99]	1	7 years	M	Rash and bilateral knee pain	Restrictive diet	Normal
Liuzzo Scorpo et al. [100]	1	3 years	F	Petechiae, follicular hyperkeratosis on the limbs, and bleeding gums	Restricted diet	Autism
Fickrey et al. [101]	1	9 years	M	Limping	Restricted diet	Normal
Listernick et al. [102]	1	15 years	M	Progressive gingival hyperplasia and 2-week history of refusal to bear weight, easy bruising, pallor, and jaundice	Restricted diet	Trisomy 21
Kow et al. [103]	1	9 years	M	Unexplained progressive bilateral lower limbs, generalized weakness and pain for 2 months	Restricted diet	Normal
Ben Ahmed et al. [104]	1	6 years	M	Limping, hemorrhagic syndrome, arthritis and weakness	Unbalanced diet	Autism
Subhash and Santosh [105]	1	7 years		Obesity, bilateral symmetrical lower limb pain, refusal to bear weight and inability to move his lower limbs; hyperkeratotic skin lesions and perifollicular hemorrhages	Not reported	No
Iamopas et al. [106]	106 children from 2003 to 2016	44.65 months ± 30.50 months	F 2.2/M 1	The common manifestations were refusal to walk, tenderness, and swelling at the lower extremities	Restrictive diet prevalently	44 cases autism, cerebral palsy, neurodevelopmental delay
Gupta et al. [107]	1	6 years, 3 years 5 years	M, M, M	All the children had irritability, tenderness to touch, and allowed only a limited movement across the joints owing to pain	Restrictive diet	2 developmental delay, 1 normal
Rittatore et al. [108]	1	11 years	M	Skeletal manifestations	Restrictive diet	ARFID
Semenetz et al. [109]	1	14 years	M	Hip pain and inability to walk	Restrictive diet	Normal
Biswas et al. [110]	1	6 years	M	Pain legs, limping	Picky eater	Noonan syndrome developmental delay
Frade et al. [111]	1	10 years	M	Disabling bone pain, mainly in the lower, associated with bicytopenia (anemia and leukopenia)	Restrictive diet	No
Masci et al. [112]	8 (from 2016 to 2021)	Median age 3.7 years	The majority (87%) were males	Mucocutaneous involvement was observed in 75% cases; microcytic anemia and elevated inflammatory markers were common laboratory findings	Restrictive diet	4: Developmental normal; 4: No
Monroig-Rivera et al. [113]	1	29 months	M	Skeletal manifestations	Restrictive diet	Autism
Goldfarb et al. [114]	1	5 years	M	Unremitting leg pain and progressive difficulty in bearing weight	Restrictive diet	Autism
Chaluvaraj et al. [115]	1	Young child	M	Gingival bleeding	Restricted diet	Learning difficulty: Talking difficulty
Patel et al. [116]	1	10 years	M	Worsening left knee pain and swelling, intermittent headaches, and diarrhea	Garlic bread, plain wheat-based noodles, and soy milk for the last 3 years	No

## 4 Discussion

Vitamin C is an essential, water-soluble micronutrient naturally present in citrus fruits and vegetables such as red pepper, potatoes, cabbage, tomatoes, and others. In humans, vitamin C plays an important role as co-factor in the synthesis of catecholamines, collagen, cortisol, neurotransmitters, peptide hormones, the immune cells functions, the maintenance of endothelial vasodilation and barrier, and the iron and acid folic metabolism [1,2,117,118]. Due to its antioxidative action vitamin C works as scavenger of reactive oxygen species and inhibits proinflammatory cytokines. The role of vitamin C in synthesizing collagen is relevant as the collagen is a vital structural protein essential for maintaining the integrity and strength of connective tissues throughout the body. The metabolism of vitamin C occurs in different stages: (a) uptake in the intestine by the sodium-dependent vitamin C transporter 1 (SVCT1), (b) free filtration in the kidneys and reabsorption in the proximal tubule via SVCT1, (c) uptake to the cells mediated by the sodium-dependent vitamin transporter 2 (SVCT2), and (d) and urinary excretion [118]. Symptoms of vitamin C insufficiency appear within 4–12 weeks by the reduced supply. In children, factors predisposing to scurvy include infant feeding habits with limited access to fruits and vegetables, eating disorders such as anorexia nervosa, picky eater, avoidant restrictive food intake disorder, and social isolation. Iron-overload conditions, food allergies, and malabsorption illnesses such as inflammatory bowel diseases, cystic fibrosis, and coeliac disease may create the basis for an incorrect absorption of vitamin C and onset of scurvy [1]. Looking at the gender it appears clear the high prevalence of scurvy in males. These data are in accordance with other reviews of the literature [18,19] and with our case series here reported (ratio 2F/8M). We believe that the gender distribution with high preponderance of males, could be partly due to a volumetric dilution effect due to the higher fat-free mass of males and also due to the high number of scurvy subjects affected by ASD a disorder which tends to show a selective and restricted food interest [5,19].

The clinical spectrum of scurvy is wide and includes musculoskeletal disturbances, mucocutaneous lesions, or systemic organs involvement. Each of one of these clinical manifestations can mimic several disorders such as autoimmune diseases, infections, hematologic disorders, and tumors [119]. In children, the initial symptoms of scurvy are unspecific with malaise, tiredness, and listlessness, as the disorder proceeds various clinical manifestations as bleeding gums, easy bruising, skin rashes, keratosis pilar, delayed wound healing, muscle and joint pains, and corkscrew strands hair which manifests with twisted or coiled shaft hairs, a sign considered diagnostic for scurvy. Other cutaneous manifestations include phrynoderma, perifollicular hemorrhages and purpura, edema of the lower extremities, and splinter hemorrhages [117]. Another sign suggestive for the diagnosis of scurvy is represented by the “scorbutic rosary,” which is characterized by costo-chondral separation with the cartilaginous positions of the ribs displaced posteriorly while sharp anterior ends of the bony ribs protrude anteriorly. This sign must be differentiated by “rachitic rosary” which is typical of children with rickets [1]. In the absence of a correct treatment, the disorder may proceed with involvement of various organs. Vascular fragility is cause of joint swelling, hemarthrosis, subperiosteal hematomas, and subconjunctival hemorrhages. In the further untreated clinical phase, bone fractures may be reported and various internal body organs may be involved [2,3]. Diagnosis of scurvy is based on medical history, clinical examination, radiographical examinations, laboratory analysis including blood vitamin C level, and effective and prompt replay to the treatment with vitamin C. A low plasma vitamin C level of less than 0.2 mg/dL is suggestive for the diagnosis of scurvy. Deficit of vitamin C is often associated with unappropriated blood level of other useful nutrients, vitamins, and minerals. For this reason, it appears useful to check in affected children of scurvy the possible lack of others factors such as vitamin B12, folate, calcium, and zinc. Hematologic examination for possible presence of anemia should be checked. Differential diagnosis may involve a range of pathological disorders according to various symptoms presented by the children with scurvy. It is important to exclude hematologic disorders as, leukaemia, immune thrombocytopenic purpura, Henoch-Schonlein purpura, disorders which are cause of malnutrition and undernutrition, celiac disease, cystic fibrosis, bone disorders like osteomyelitis and septic arthritis, and in some occasion scurvy may also mimic a pseudo-paralysis.

Treatment consists of vitamin C supplementation. For children, recommended doses of 100–300 mg of ascorbic acid given daily according to the age and the grade of clinical involvement. The treatment should be continued till complete recovery. Then it is important to recommend parents to include citrus fruits in the diet of the children. Prognosis is linked to a prompt diagnosis and treatment and to the incidental underlying factors which have determined the loss of vitamin C. In children, without severe underlying disorder, the prognosis is benign after accurate diagnosis and treatment which leads to rapid recovery. The great relevance is to avoid the relapses involving parents and caregivers on the correct diet treatment and to treat the various disorders which in some case may have caused the lack of vitamin C.

The interest on scurvy of pediatric community came back because of the observation in the current clinical practice of the presence of this disorder. In contrast to the past, it should be suspected not only in children with neurodevelopmental disabilities and low income population, but also – and may be more – in typically developing children with home-routine diet devoid of fruits and vegetables. Of course, in developmental countries it is not easy to suspect a disorder linked to nutritional deficiency. Through the review of the literature, we noted that the term “picky eater” appears in 2019 in a report of child affected by scurvy [73]. This term refers to children who choose to take very restrictive and selective diets mainly based on carbohydrates. In the large majority of these cases, the consumption of vegetables and fruit is not present.

Neurodevelopmental disorders play an important role in the onset of scurvy. In this systematic review, a high number of children presented with ASD, neurodevelopmental disorders, cerebral palsy, and other neurologic diseases. Dysfunctional feeding behaviors might be particularly critical for children affected by ASD who are more likely to avoid food compared to typically developing children due to their sensory issues and their restricted interests and behaviors [4]. In the present case-series neurobehavioral disorders were found in two children one affected by ASD and another by avoidant/restrictive food intake disorder (ID-06, ID-07). With regard to cases ID-01, ID-02, ID-03, ID-05, and ID-08 we noted on clinical history that parents revealed to have refused to give citrus fruits to their children on the prejudice diffuse in some of lower classes of the Sicilian population that oranges and lemon can cause bladder infections or when mixed to milk to cause toxic gastrointestinal effects. This hypothesis may contribute to explain as scurvy is still present in Sicily, the recognized “country of citrus fruits.” Interestingly, a brief report of Gilley et al. [120] published in 2024 (non-included in this criteria systematic review), reported 47 patients with a diagnosis of scurvy, 49% of whom had a neurodevelopmental disorder. In addition, Gilley et al. [120] reported that 16of the 47 patients complained of musculoskeletal symptoms: 3 of the 16 individuals had moderate or severe malnutrition whereas 3 had overweight or obesity, underling the thesis that malnutrition and obesity can coexist in scurvy, but obesity can be an unfavorable factor for the diagnosis. Obesity is never synonym of a good and complete diet, thus deficiency and scurvy should be also suspected in overweight children. These data are also confirmed in another casuistic published by Iamopas et al. [106], in which the nutritional status was characterized by obesity in 12% of the patients, normal in 70% and wasting in 18%.

Most article reported a late diagnosis because of various factors. First, because scurvy is a neglect disease, considered as a disorder of the past. Second, because of the wide spectrum of symptoms, rarely presented at the same time and in the same patient. Third, because laboratory examinations and radiological investigations are rarely helpful. The report of Iamopas et al. [106] showed a misdiagnosis in 74 cases (69%).

## 5 Conclusions

Vitamin C is an essential nutrient for humans with relevant effects on functionality of various organs and systems including heart, lung, kidney, brain, blood, and immune defence [121]. Vitamin C is involved in many biochemical processes in human body and its low level is cause of the scurvy, a disorder, which is again present in large part of the world. However, the disorder is not common in the country with elevated socio-economic status, and it is present in underdeveloped area with children malnourished or manifesting underlying disorders which prevent absorption of vitamin C. Scurvy may be also found in children with overweight and in those whose parents show false prejudice that citrus fruits may cause cystitis or toxic effect when mixed with milk. Classic oral manifestations of gingivitis with bleeding and swelling gums are the suggestive signs for a precocious diagnosis of scurvy [122] as well as limping, refusal to walk, musculoskeletal complains, and lower limbs pain are all signs that are indicative for a correct and rapid diagnosis. In children, the disorder may have a good prognosis in the absence of underlying disorders and treatment with adequate dose of vitamin C may lead to rapid improvement and recovery.

Our experience and the systematic review of literature of the last two decades confirmed that scurvy is an historical but not vanished disorder. Family behavior normally permits a functional development of feeding in children. The child’s nutritional well-being extends from school to home: and, as pediatricians, we underline the need of a nutritional educational program for parents that starts from institutions.
